# Higher leukocyte count predicts 3-month poor outcome of ruptured cerebral aneurysms

**DOI:** 10.1038/s41598-018-23934-x

**Published:** 2018-04-11

**Authors:** Pei-Sen Yao, Guo-Rong Chen, Xue-Ling Xie, Huang-Cheng Shang-Guan, Jin-Zhen Gao, Yuan-Xiang Lin, Shu-Fa Zheng, Zhang-Ya Lin, De-Zhi Kang

**Affiliations:** 1 Department of Neurosurgery, The First Affiliated Hospital of Fujian Medical University, Fuzhou, China; 2 Department of Critical Care, The First Hospital of Fuzhou, Fuzhou, China; 3 Institute of Neurology, The First Affiliated Hospital of Fujian Medical University, Fuzhou, China

## Abstract

It is not fully established whether leukocyte can predict the poor outcome for ruptured cerebral aneurysms (CA) or not. Here, we retrospectively analyzed the clinical data of 428 patients with ruptured CA between 2010 and 2015. Patients’ demographic data, including gender, age, history of smoking, alcohol, hypertension, diabetes and hypercholesterolemia, Hunt-Hess and Fisher grade, occurrence of hydrocephalus, aneurysm location, time to surgery, delayed ischemic neurological deficit (DIND) and peak leukocyte of blood test from day 1 to 3 after aneurysmal rupture were recorded and analyzed. In the multivariable analysis model, gender, Fisher grade, time to surgery and hydrocephalus were not relevant to poor outcome. However, Hunt-Hess grade, DIND and preoperative leukocyte count (>13.84 × 10^9^/L) were significantly associated with adverse outcome. The respective increased risks were 5.2- (OR 5.24, 95% CI 1.67–16.50, p = 0.005), 6.2-(OR 6.24, 95% CI 3.55–10.99, p < 0.001) and 10.9-fold (OR 10.93, 95% CI 5.98–19.97, p < 0.001). The study revealed that Hunt-Hess grade, DIND and preoperative leukocyte count (>13.84 × 10^9^/L) were independent risk factors for poor outcome of ruptured CA at 3 months. Higher leukocyte count is a convenient and useful marker to predict 3-month poor outcome for ruptured CA.

## Introduction

The prevalence of cerebral aneurysms (CA) affects 3–5% of the general population^1^, and subarachnoid hemorrhage caused by ruptured CA is a fatal or disabling stroke in these people. It was reported that several risk factors (such as older age, poor Hunt & Hess grade, higher Fisher grade, hypertension, hydrocephalus, hyperglycemia, excess weight and time to surgery) could lead to and predict the poor outcome for ruptured CA^2,3^.

It has been found that leukocytes are associated with the adverse prognosis in glioma^4,^ coronary artery disease^5^, abdominal aortic aneurysms^6^. Pozzilli^7^ and Yoshimoto^8^ reported that higher leukocyte counts in peripheral blood were acute systemic inflammatory response following subarachnoid hemorrhage, and the number of leukocytes reflected the degree of brain tissue damage and predicted further secondary brain injury. Recent studies suggested that elevated leukocyte count predicted the adverse outcome in ischemic and hemorrhagic stroke^9,10^. Previous studies have shown a relationship between leukocytosis and cerebral aneurysm, but it is still not fully established whether leukocyte can predict the poor outcome for ruptured CA or not. A common limitation in these studies was a relatively small number of patients (less than 172) treated by surgical clipping^11–14^. In order to test the hypothesis that preoperative higher leukocyte count is associated with and predict the poor outcome at 3 months, we retrospectively analyzed the clinical data of a relatively large number of patients with ruptured CA treated with microsurgical clipping.

## Methods

All procedures performed in this study involving human participants were in accordance with the 1964 Helsinki declaration and approved by the ethics committee of First Affiliated Hospital of Fujian Medical University. Informed consent was obtained from all individual participants included in the study. Four hundred and twenty eight patients with ruptured CA between 2010 and 2015 were collected. Gender, age, history of smoking, alcohol, hypertension, diabetes, hypercholesterolemia, Hunt-Hess and Fisher grade on admission, acute hydrocephalus, aneurysm location, time to surgery, delayed ischemic neurological deficit (DIND), peak leukocyte count of blood test from day 1–3 after ruptured were recorded. Patients were eligible for enrollment if following criteria were met: (1) Subarachnoid hemorrhages were confirmed by Computed Tomography (CT). Then the CA were diagnosed by computed tomography angiography (CTA) or digital subtraction angiography (DSA); (2) All aneurysms underwent microsurgery, and postoperative CTA and/or DSA were performed. The exclusion criteria were: (1) Ruptured CA were detected over 3 days; (2) The patients presented with herniation, or were associated with the other cerebrovascular diseases (such as arteriovenous malformations, arteriovenous fistula, and moyamoya disease) and brain tumor; (3) The patients were associated with fever (over 38 °C), infection, inflammatory process, or received any medical treatment that might have an influence on the leukocyte count in peripheral blood.

### Management of ruptured CA

CTA or DSA was performed during the first 24 hours after admission. After being confirmed, aneurysms were repaired with microsurgical treatment. After aneurysmal clipping, the patients were managed with a traditional treatment of aneurysmal subarachnoid hemorrhage, including prevention of cerebral vasospasm, improving cerebral blood flow, prevention of stress ulcers and nutritional support. CT scanning for detecting postoperative complications has been performed within 24 hours after surgical treatment. The neurological outcome was assessed at the 3-month follow-up and categorized according to the patients’ modified Rankin Scale (mRS) score. A favorable outcome was defined as mRS 0–3, while a poor outcome as mRS 4–6.

### Statistical Analysis

All the statistical analyses were performed using SPSS version 22.0 (IBM Corp., Armonk, NY, USA). The significance of differences in continuous data was determined with one-way variance (ANOVA) and Student’s t test. Qualitative data were compared using a chi-squared test (χ^2^ test) or Fisher’s exact test. Multivariable analysis logistic regression analyses included all variables significance level at p < 0.15 in univariate analysis. For inclusion in the multivariable analysis model, age was dichotomized as “less than 60 years” and “more than 60 years”^15^, Hunt-Hess grade as “low grade (Grade I-III)” and “high grade (Grade IV-V)”, Fisher grade as “low grade (Grade 1, 2, 3)” and “high grade (Grade 4)”, surgical time as “less than 3 days” and “more than 3 days”, leukocyte counts as “≤ optimal cutoff value” and “> optimal cutoff value”. P < 0.05 was deemed statistically significant. A MedCalc 15.2.2 (MedCalc Software, Mariakerke, Belgium) was used to generate the receiver operating curve (ROC) and analyze the specificity, sensitivity, negative predictive values of leukocytes and positive predictive values of leukocytes for mRS.

## Results

Four hundred and twenty eight patients were included in the retrospective study according to the upper inclusion and exclusion criteria. The clinical characteristics of these patients with ruptured CA are shown in Table 1. The mean age of 307 (72%) patients in the good outcome group was55.8 ± 10.6 years, and 121 (28%) patients with a poor outcome was 56.6 ± 10.8 years (p > 0.05). The univariate analysis indicated there were significant differences were detected in gender, Fisher grade, hydrocephalus, time to surgery, DIND, leukocyte count between favorable and poor groups (p < 0.05). One hundred and ninety-four patients (45%) were male, 234 (55%) were female, the difference of the gender distribution between the two groups was statistically significant (p < 0.05). There were no significant statistical differences in the number of the patients with history of smoking, alcohol, hypertension, diabetes, hypercholesterolemia between the two groups (p > 0.05). The aneurysm location between the two groups was not significantly statistical difference (p > 0.05). There was no significant difference of preoperative rebleeding between the two groups (6 patients in the favorable and 4 in the poor outcome group) (p > 0.05).

**Table 1:** Characteristics of 428 consecutive patients with ruptured CA.

Variable	**mRS (0–3)**	**mRS (4–6)**	**p-value** ^**a**^
	**Good outcome (n = 307)**	**Poor outcome (n = 121)**	
**Age (years)**			
M ± SD	55.8 ± 10.6	56.6 ± 10.8	0.444
Age > = 60, N (%)	105 (34)	49 (40)	0.263
Age <60, N (%)	202 (66)	72 (60)	
**gender, N (%)**			<0.001
Male	118 (38)	76 (63)	
Female	189 (62)	45 (37)	
Smoking, N (%)	54 (18)	20 (17)	0.887
Alcohol, N (%)	44 (14)	17 (14)	1.000
Hypertension, N (%)	157 (51)	67 (55)	0.453
Diabetes, N (%)	51 (17)	16 (13)	0.461
Hypercholesterolemia, N (%)	119 (39)	54 (45)	0.276
**Hunt & Hess grade on admission, N (%)**			0.065
Grade I-III	236 (77)	82 (68)	
Grade IV-V	71 (23)	39 (32)	
**Fisher grade on admission, N (%)**			<0.001
Grade 1, 2, 3	230 (75)	50 (41)	
Grade 4	77 (25)	71 (59)	
Hydrocephalus, N (%)	58 (19)	42 (35)	0.001
**Aneurysm location, N (%)**			0.791
ACA	18 (5)	6 (4)	
AcomA	96 (27)	40 (29)	
MCA	98 (27)	31 (22)	
ICA/PcomA	142 (40)	60 (43)	
PCA	4 (1)	1 (1)	
**Time to surgery, N (%)**			<0.001
<72 h	86 (28)	57 (47)	
>72 h	221 (72)	64 (53)	
DIND	80 (26)	82 (68)	<0.001
Preoperative rebleeding, N (%)	6 (2)	4 (3)	0.478
Leukocyte count (*10^9^/L)	10.34 ± 3.23	15.24 ± 5.47	<0.001

mRS = modified Rankin Scale; ACA = anterior cerebral artery; ACoA = anterior communicating artery; MCA = middle cerebral artery; ICA = Internal Carotid Artery; PcomA = posterior communicating artery; PCA = posterior cerebral artery; DIND = delayed ischemic neurological deficit.

**Figure 1:**
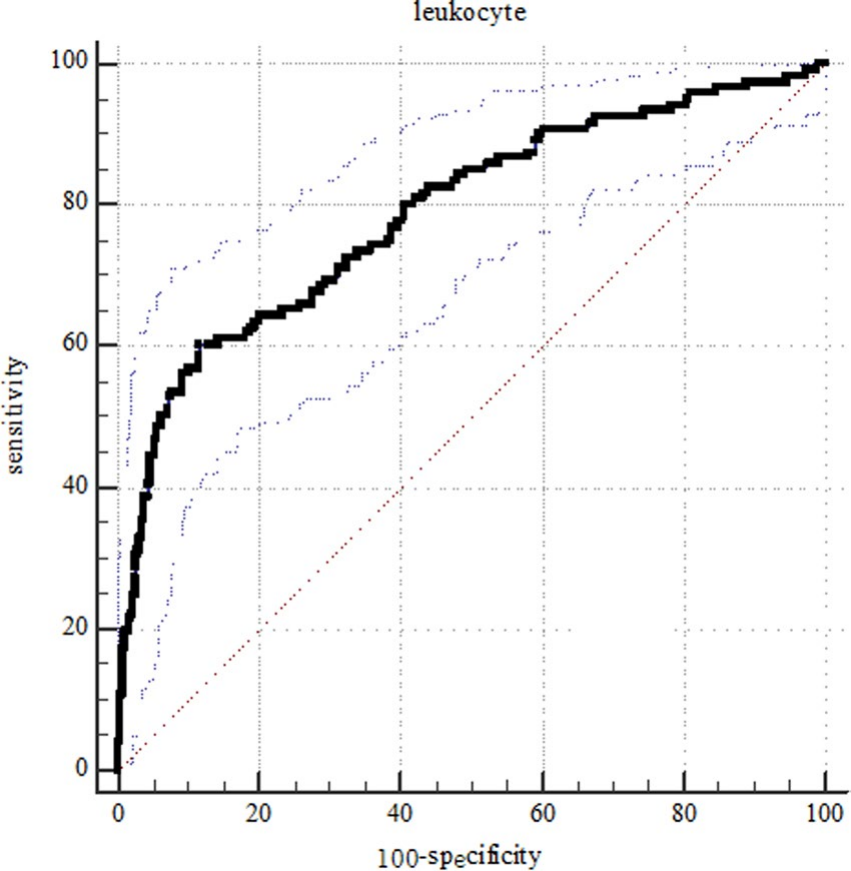
Predictive values of leukocyte count for the 3- month modified Rankin Scale(mRS) >3 Area under curve 0.787 (95% confidence interval [CI], 0.745–0.825; p < 0.001).

The median peak leukocyte count (×10^9^/L) in the good outcome group (10.34 ± 3.23) was lower than that in the poor outcome group (15.24 ± 5.47). The receiver operating characteristic (ROC) curve is shown in Fig. 1. The optimal cutoff value for leukocyte as a predictor for 3-month status was determined as 13.84 (×10^9^/L) in the ROC curve (sensitivity was 60.3%, and the specificity was 88.3%). In addition, the mean of leukocyte count in patients with Hunt-Hess grade IV-V (16.98 ± 6.54) was higher than that in Hunt-Hess grade I-III (11.33 ± 4.12), the difference was statistically significant (p < 0.001).

Multivariate logistic regression model for mRS was built, and we included all preoperative variables (gender, Fisher grade, time to surgery, hydrocephalus, DIND and leukocyte count) with a significance level at p < 0.15 in the univariate analysis. The results revealed that Hunt-Hess grade, Fisher grade, hydrocephalus, DIND and leukocyte count were associated with 3-month poor outcome. After adjustment for potential confounding variables, gender, Fisher grade, time to surgery and hydrocephalus were not relevant to poor outcome, while Hunt-Hess grade, DIND and preoperative leukocyte count (greater than 13.84 × 10^9^/L) remained significantly associated with adverse outcome (Table 2), the respective increased risks were 5.2-[odd ratio (OR)5.24, 95% confidence interval (CI)1.67–16.50, p = 0.005], 6.2-(OR6.24, 95% CI 3.55–10.99, p < 0.001) and 10.9-fold (OR10.93, 95% CI 5.98–19.97, p < 0.001) (Table 2).

**Table 2:** Predictors of poor outcome for ruptured CA in multivariate model.

Independent Variable	Unadjusted		Adjusted	
	OR (95%CI)	P value	OR (95%CI)	P value
Gender	0.948 (0.61–1.46)	0.811	0.81 (0.46–1.43)	0.472
Hunt & Hess grade	12.41 (4.93–31.25)	<0.001	5.24 (1.67–16.50)	0.005
Fisher grade	3.41 (2.20–5.30)	<0.001	1.61 (0.87–1.96)	0.128
Hydrocephalus	2.28 (1.43–3.66)	0.001	1.59 (0.83–3.06)	0.163
Time to surgery	0.80 (0.52–1.25)	0. 330	1.08 (0.60–1.93)	0.805
DIND	5.97 (3.77–9.44)	<0.001	6.24 (3.55–10.99)	<0.001
Leukocyte >13.84*10^9^/L	11.45 (6.92–18.94)	<0.001	10.93 (5.98–19.97)	<0.001

OR = odds ratio; CI = confidence interval.

Multivariate logistic regression model for DIND was also built to include the preoperative variables with a significance level at p < 0.15 in the univariate analysis. The results revealed that Hunt-Hess grade IV-V and a serum leukocyte count greater than 13.84 × 10^9^/L were associated with DIND, the increased risks were 2.4-fold (OR2.41, 95% CI 1.40–5.60, p = 0.040) and 1.7-fold (OR1.73, 95% CI 1.08–2.78, p = 0.023) respectively (Table 3).

**Table 3:** Predictors of DIND for ruptured CA in multivariate model.

Independent Variable	Unadjusted		Adjusted	
	OR (95%CI)	P value	OR (95%CI)	P value
Gender	1.24 (0.84–1.86)	0.277	1.29 (0.86–1.96)	0.215
Hunt & Hess grade	3.61 (1.64–7.92)	0.001	2.41 (1.40–5.60)	0.040
Fisher grade	1.87 (1.23–2.82)	0.031	1.35 (0.84–2.15)	0.214
Hydrocephalus	2.28 (1.43–3.66)	0.001	1.35(0.82–2.20)	0.237
Time to surgery	1.02 (0.68–1.55)	0.915	1.11 (0.72–1.71)	0.634
Leukocyte >13.84*10^9^/L	2.12 (1.37–3.30)	0.001	1.73 (1.08–2.78)	0.023

OR = odds ratio; CI = confidence interval.

## Discussion

The incidence of poor outcome reported in previous studies ranged from 20 to 40%^16,17^. In this 6-year review of 428 consecutive patients with ruptured CA, the risk factors including gender, Fisher grade on admission, time of surgery, hydrocephalus and preoperative higher leukocyte count between the two groups are statistically significant, which may be associated with 3-month poor outcome. Previous studies indicated that hydrocephalus and time to surgery were associated with poor outcome of ruptured CA^18,19^. However, in the logistic regression models, it was found that only three variables (Hunt-Hess grade, DIND and preoperative higher leukocyte count) were the independent risk factors for predicting 3-month poor outcome, while female gender, higher Fisher grade, hydrocephalus and time to surgery not.

Neuronal and axonal damage may occur in the pathophysiological process after aneurysmal rupture^20^, and a deterioration of the clinical neurological condition was observed. It is no doubt that poor Hunt-Hess grade on admission strongly predicted the poor outcome in aneurysmal subarachnoid hemorrhage, and our finding is consistent with prior report^21^. We found that the leukocyte count in the patients with Hunt-Hess grade I-III on admission was lower than that in Hunt-Hess grade IV-V within 3 days after aneurysm rupture. Thus, leukocyte count in peripheral blood may be associated with the severity of the acute primary injury of the brain tissue. Furthermore, our findings suggested that Hunt-Hess grade IV-V and a serum leukocyte count greater than 13.84 × 10^9^/L in this study were associated with developing DIND, which was related to poor outcome^22–24^.

As we know, the increased leukocyte count may be the result of acute phase response and reflects the severity of disease and tissue inflammatory response. In 1974, Neil-Dwyer and Cruichshank^14^ firstly revealed that higher leukocyte count was associated with the unfavorable outcome of ruptured CA. On the contrary, Spallone^12^ found no relationship between the admission higher leukocyte count and ischemic complications. Therefore, studies assessing the role of leukocyte in the adverse outcome of ruptured CA have yielded conflicting conclusions^12,13^.

Interestingly, the main finding of our study revealed that higher leukocyte count before microsurgical treatment was an independent risk factor for poor outcome of ruptured CA at 3 months. Previous results showed that the inflammatory reaction occured after cerebral aneurysm rupture. Leukocytes in peripheral blood would be recruited within the first several hours because of the blood released into the subarachnoid space^25^, which would increase intracranial pressure and lead to early brain injury (EBI). Accompanying with releasing chemotactic factor into the central nervous system and an increase in the number of leukocytes in peripheral blood, the leukocyte will be recruited in the brain microvasculature^26^, which is associated with blood–brain barrier disruption and basal lamina degradation^27^. As a result, brain edema might occur or be aggravated together with neuroinflammation-promoted apoptosis in sympathetic neurons, which would further aggravate secondary brain damage^28^.

It is noteworthy that several molecular mechanisms including Angiotensin II (Ang II)^29^, myeloperxidase^30^, NF-κB^31^, cyclooxygenase-2 (COX-2) and microsomal prostaglandin E2 synthase-1 (mPGES-1)^32^, were associated with the adverse outcome after aneurysm rupture, but the detailed mechanisms of leukocyte in cerebral aneurysm remain obscure. A growing body of evidence suggested that leukocyte infiltration in the aneurysmal wall contributed to the formation, growth and rupture of cerebral aneurysm, which is increasingly recognized as an inflammatory process^33^. Furthermore, histopathological findings of aneurysmal wall indicated that the more leukocytes were observed, the more fragile aneurysms became^34^.

Several inflammatory mediators synthesized and/or expressed by leukocyte, such as IL-1β, IL-6, TNF-α, TLR4, and matrix metalloproteinases, will further aggravate the local inflammatory response which is closely related to an adverse outcome^35^. Maiuri, *et al*.^36^ and McGirt, *et al*.^11^ came to a common conclusion that leukocyte counts had a high prognostic value in predicting cerebral vasospasm which was associated with neurological deterioration and poor outcome. Inhibiting the inflammatory response and reducing leukocyte activity was highly beneficial in limiting secondary brain injury and increasing survival after aneurysm rupture^37^.

### Limitation

In the retrospective study, the other biomarkers, such as Erythrocyte Sedimentation Rate (ESR), C Reactive Protein (CRP), complement C3 and C9, were not included in the study. Further other biomarkers examination should be performed.

## Conclusion

Hunt-Hess grade, DIND and preoperative higher leukocyte count (>13.84 × 10^9^/L) before microsurgical treatment were independent risk factors for poor outcome of ruptured CA at 3 months. Higher leukocyte count before microsurgical treatment is a convenient and useful marker to predict poor outcome of ruptured cerebral aneurysm at 3 months.
