# Author Correction: Higher leukocyte count predicts 3-month poor outcome of ruptured cerebral aneurysms

**DOI:** 10.1038/s41598-018-31339-z

**Published:** 2018-08-24

**Authors:** Pei-Sen Yao, Guo-Rong Chen, Xue-Ling Xie, Huang-Cheng Shang-Guan, Jin-Zhen Gao, Yuan-Xiang Lin, Shu-Fa Zheng, Zhang-Ya Lin, De-Zhi Kang

**Affiliations:** 1 Department of Neurosurgery, The First Affiliated Hospital of Fujian Medical University, Fuzhou, China; 2 Department of Critical Care, The First Hospital of Fuzhou, Fuzhou, China; 3 Institute of Neurology, The First Affiliated Hospital of Fujian Medical University, Fuzhou, China

Correction to: *Scientific Reports* 10.1038/s41598-018-23934-x, published online 11 April 2018

The original version of this Article contained an error in the Abstract and Results.

In the Abstract, “The respective increased risks were 5.2- (OR5.24, 95% CI 1.67–16.50, p = 0.005), 6.2-(OR 6.24, 95% CI 3.55–10.99, p < 0.001) and 10.9-fold (OR 9.35, 95% CI 5.98–19.97, p < 0.001).”

now reads,

“The respective increased risks were 5.2- (OR5.24, 95% CI 1.67–16.50, p = 0.005), 6.2-(OR 6.24, 95% CI 3.55–10.99, p < 0.001) and 10.9-fold (OR 10.93, 95% CI 5.98-19.97, p < 0.001).”

In the Results, “After adjustment for potential confounding variables, gender, Fisher grade, time to surgery and hydrocephalus were not relevant to poor outcome, while Hunt-Hess grade, DIND and preoperative leukocyte count (greater than 13.84 × 10^9^/L) remained significantly associated with adverse outcome (Table 2), the respective increased risks were 5.2-[odd ratio (OR)5.24, 95% confidence interval (CI)1.67–16.50, p = 0.005], 6.2-(OR6.24, 95% CI 3.55–10.99, p < 0.001) and 10.9-fold (OR9.35, 95% CI 5.98–19.97, p < 0.001) (Table 2).”

now reads:

“After adjustment for potential confounding variables, gender, Fisher grade, time to surgery and hydrocephalus were not relevant to poor outcome, while Hunt-Hess grade, DIND and preoperative leukocyte count (greater than 13.84 × 10^9^/L) remained significantly associated with adverse outcome (Table 2), the respective increased risks were 5.2-[odd ratio (OR)5.24, 95% confidence interval (CI)1.67–16.50, p = 0.005], 6.2-(OR6.24, 95% CI 3.55–10.99, p < 0.001) and 10.9-fold (OR10.93, 95% CI 5.98–19.97, p < 0.001) (Table 2).”

These errors have now been corrected in the PDF and HTML versions of the Article.

